# Two-Axis Continuous Distractor for Mandibular Reconstruction

**DOI:** 10.3390/bioengineering9080371

**Published:** 2022-08-06

**Authors:** Shahrokh Hatefi, Milad Etemadi Sh, Javad Alizargar, Venous Behdadipour, Khaled Abou-El-Hossein

**Affiliations:** 1 Ultra-High Precision Manufacturing Laboratory, Department of Mechatronics Engineering, Faculty of Engineering, the Built Environment and Technology, Nelson Mandela University, Port Elizabeth 6000, South Africa; 2 Department of Oral and Maxillofacial Surgery, Dental Implants Research Center, Dental Research Institute, School of Dentistry, Isfahan University of Medical Sciences, Isfahan 81746-73461, Iran; 3 Research Center for Healthcare Industry Innovation, National Taipei University of Nursing and Health Sciences, Taipei 112, Taiwan; 4 School of Nursing, National Taipei University of Nursing and Health Sciences, Taipei 112, Taiwan; 5 College of Agricultural Engineering, Isfahan University of Technology, Isfahan 84156-83111, Iran

**Keywords:** maxillofacial reconstruction, bone regeneration, distraction osteogenesis

## Abstract

The application of Distraction Osteogenesis (DO) techniques in the reconstruction of skeletal deficiencies is a relatively new topic in the fields of oral and maxillofacial surgeries. In many reconstruction applications, using DO is the preferred technique, as opposed to conventional reconstruction techniques, as there are more advantages and fewer side effects when it is used. The first generation of DO devices is made up of manual distractors that can apply an intermittent distraction force to the bone segment during the distraction process. Manual DO techniques have shown the functionality of the DO technique. Further research has recently been performed on the development of automatic devices for generating a controlled continuous force. However, the existing automatic techniques have limitations, and are yet to be used in reconstruction applications in humans. There is still a gap between the developed techniques and an ideal distractor to be used in mandibular reconstruction surgeries. In this research, a two-axis continuous distractor is proposed for use in mandibular reconstruction applications. The proposed distractor can generate two continuous distraction forces that can be applied to two independent distraction vectors. The proposed device can perform the standard distraction process using the predetermined distraction factors. The control system has a high positioning accuracy and resolution in controlling the position of the intra-oral end effectors while applying two continuous forces for moving the bone segment. The proposed two-axis continuous distractor meets the current requirements, and can be used as an ideal continuous DO device for different mandibular reconstruction applications.

## 1. Introduction

The application of medical robotics and advanced control systems plays an important role in the development of novel surgical techniques and reconstruction methods for tissue engineering applications, as well as regenerative medicine for the reconstruction of bone deficiencies in different body zones [1]. The results of recent studies show that a large number of patients with mandibular deficiency, mandibular prognathism, and asymmetry problems in the oral and maxillofacial areas seek reconstruction treatments [2,3]. Different surgical techniques and reconstruction methods have been developed for use in Mandibular Reconstruction Applications (MRAs), including vascularized flaps, prosthetics, bone grafting, Distraction Osteogenesis (DO), and tissue engineering techniques [4]. Among the developed techniques for MRAs, DO is the first choice for many reconstruction conditions in the oral and maxillofacial areas [5].

**Figure 1:**
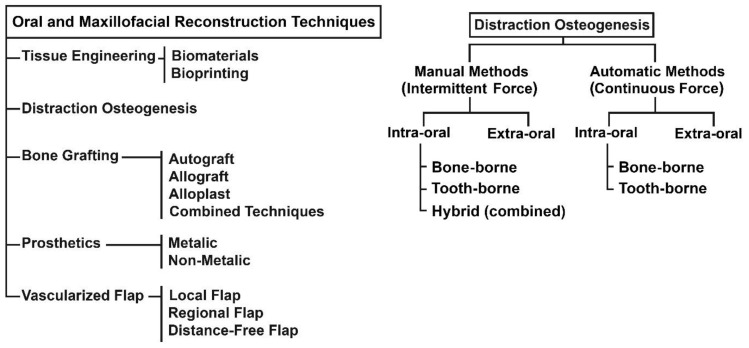
Oral and maxillofacial reconstruction techniques and the existing DO methods.

The DO technique can be used in the reconstruction of different oral and maxillofacial deficiencies, including acquired/congenital bone loss, congenital growth retardation, congenital malformations, post-traumatic injuries, and post-tumor-resection [6,7,8,9,10]. This technique is known as a reconstruction method without the need for a bone graft [11,12,13]. It has shown promising results in terms of improving the conditions of the treatment and the quality of the outcome, while also making the treatment time shorter. By using DO in MRAs, high-quality bone tissue, along with the surrounding soft tissue, is regenerated, with better postoperative stability and a more predictable healing process [14,15]. The DO technique can also reduce the negative effects of the conventional reconstruction techniques on the patient, including blood loss, scar formation, pain, and postoperative complications [4]. Figure 1 shows the reconstruction techniques in the oral and maxillofacial areas, along with the existing DO methods in MRAs.

The basic science and principles of the DO were developed and introduced by Ilizarov in 1987 [16,17,18]. In MRAs, a standard DO process consists of the following steps: At first, during a surgical operation, an osteotomy line is defined, a Bone Segment (BS) is osteotomized from the defected zone, and the distractor is installed on the distraction zone, where the osteotomized BS is fixed to the moving part of the distractor. There is a latency phase after the osteotomy and installation of the distractor. In the latency phase, the regenerative phase starts, and the osteogenic cells begin regeneration and consolidation. The restoration of the bone is likely to be observed by means of callus tissue that forms around and between the segments of bone. After the latency period, the activation phase starts and the DO device moves the BS in the predetermined linear vector—called the Distraction Vector (DV)—towards the desired position. In the activation phase, the traction is applied such that there is a formation of woven bone fibers parallel to the DV. Therefore, the generated gap between the main bone part and the moving BS is filled with regenerated bone tissue. After the completion of the activation phase, the consolidation phase begins. In the consolidation phase, the distractor is deactivated, and the regenerated bone tissue consolidates. At the end of this phase, in a second surgical operation, the DO device can be removed [6,19,20].

The conventional DO devices are manual distractors with mechanical mechanisms and manual/intermittent force-generation techniques. These manual distractors are surgeon- and patient-dependent, requiring manual activation, usually performed twice daily. Distractor failure, long treatment, and non-compliance are the major drawbacks in using manual DO devices [21]. Recently, studies have been focused on the development of the next generation of DO devices and developing automatic systems for performing continuous DO in MRAs. The automatic methods can eliminate the need for patient compliance and reduce the risks and complications of DO. In automatic DO, an automatic system is used to generate a Distraction Force (DF) without the need for manual activation of the device. Furthermore, by using a continuous distractor, a higher Distraction Rate (DR) can be used, without sacrificing the quality of regenerated bone, thus shortening the treatment period. The existing continuous distractors are yet to be used in human applications [5,22,23].

**Figure 2:**
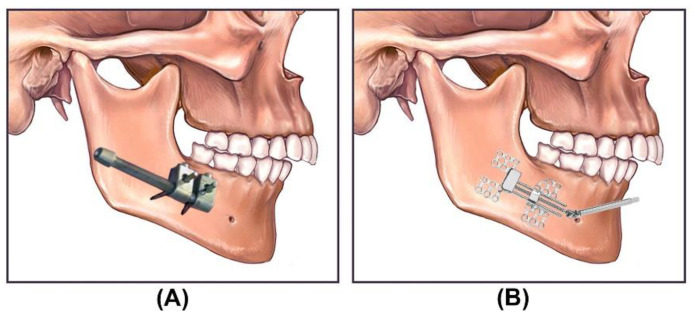
Illustration of the application of manual distractors in MRAs: (**A**) extra-oral distractor; (**B**) intra-oral distractor.

Based on the mechanism, installation technique, and the method of force transition, the developed DO devices can be categorized into two groups: intra- and extra-oral distractors. Both manual and automatic distraction techniques can be either applied intra- or extra-orally. Figure 2 illustrates the application of intra- and extra-oral distractors in MRAs. The extra-oral distractors are placed outside of the body and fixed to the bone using biocompatible mechanical fixtures. The DF is applied to the extracorporeal mechanism, and moves the BS towards the desired position through a mechanical transition mechanism. In the intra-oral DO methods, the distractor is placed inside the body—on the defected bone—to move the BS towards the desired position. Different complications and limitations are associated with the application of both manual and automatic distractors, including the size of the distractor, breakage of the distractor, tissue damage, scar formation, and pain [23,24,25,26,27]. The application of extra-oral distractors is associated with more complications and side effects compared to intra-oral distractors, including scar formation, size, visibility, infections, edema, and patients’ psychological problems [28,29,30,31].

### Distraction Osteogenesis for Mandibular Reconstruction

The conventional reconstruction techniques are gradually being replaced by novel reconstruction methods, such as DO techniques [32,33,34]. The congenital and acquired deficiencies that can be reconstructed using DO include hemifacial microsomia, mandibular body segmental defects, retrognathia, occlusal plane correction, dimensional insufficiency, micrognathia, and defects in the dimensions and position of the alveolar ridge [8,35,36,37,38,39,40,41]. Experimental studies and clinical trials show that using DO in MRAs can improve the stability of the reconstructed soft and hard tissue, and can reduce the relapse and the occurrence of neurosensory disturbance, compared to conventional mandibular reconstruction methods [42,43,44].

**Figure 3:**
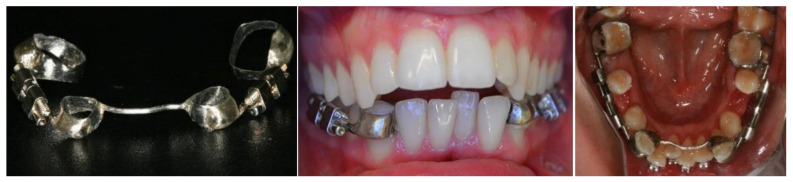
TB distractor with manual activation for reconstruction of mandibular retrognathia [45].

Generally, in a typical DO in MRAs, Bone-Borne (BB) distractors are used. In a BB distractor, the moving part of the distractor is fixed to the BS, and directly transfers the DF to the BS. Figure 2 illustrates the application of BB intra- and extra-oral distractors in MRAs. Recently, intra-oral Tooth-Borne (TB) distractors have been developed to be used in MRAs, where the generated DF is transferred to specific teeth instead of the BS. Unlike BB distractors, there is no need for a surgical operation for the installation of the TB distractor on the distraction zone. However, a surgical operation is required for the osteotomy of the BS. Figure 3 illustrates the application of a manual TB dental-anchored distractor in reconstruction of mandibular retrognathia. For the reconstruction of mandibular retrognathia, dental-anchored distractors can be used to apply the DF to the moving BS of the mandible [45]. No surgical operation is required for the installation and removal of the TB distractor. The application of dental-anchored DO is easier and more patient-friendly when compared to the BB distractors. The TB distractors can enable the execution of distraction parallel to the occlusal plane or chosen vectors [29,31,45,46,47,48]. However, there are limitations and side effects when a TB distractor is used, such as the limited Distraction Length (DL) and the orthodontic teeth movements as the DF induces bone resorption on the pressure side and bone apposition on the tension side.

**Figure 4:**
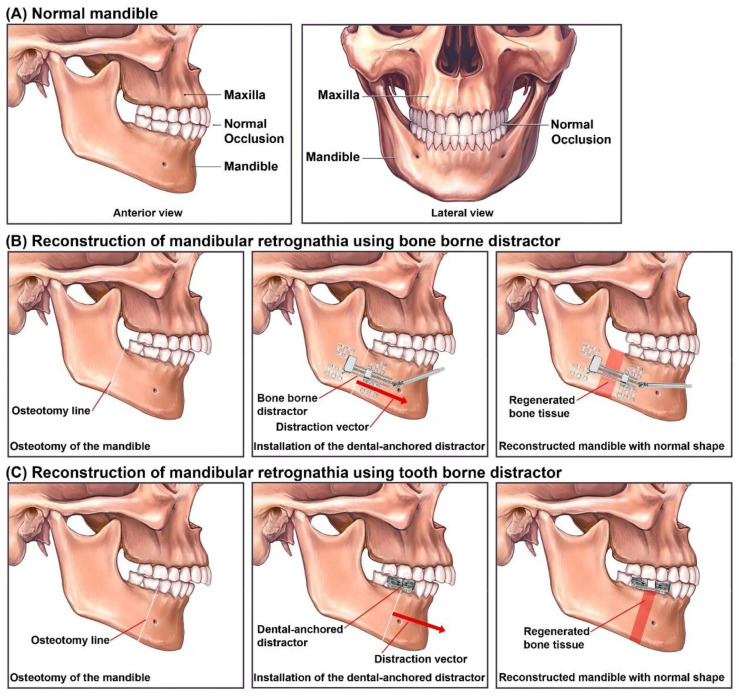
Reconstruction of mandibular retrognathia using BB and TB distractors: (**A**) Normal Mandible; (**B**) Reconstruction of mandible using BB distractor; (**C**) Reconstruction of Mandible using TB distractor.

Figure 4 illustrates the principles of a reconstruction process for treating mandibular retrognathia using BB and TB distractors. When BB distractors are used (B), in the first surgical operation, the BS is osteotomized at the desired osteotomy lines on both sides of the mandible, and two manual distractors are installed on the distraction zone, where each distractor can apply an intermittent DF in the desired DV. The osteotomy line determines the DVs. After the completion of the Distraction Process (DP), during the second surgical operation, the distractors are removed. When TB distractors are used (C), the reconstruction process starts with a surgical operation for the osteotomy of the mandible at the desired osteotomy lines on both sides of the mandible. Afterwards, the TB dental-anchored distractor is installed on the mandible and fixed to specific teeth on both the main and moving parts of the mandible. During the DP, the generated DFs are applied to both sides of the BS in the desired DVs. After the completion of the DP, the dental-anchored distractor is removed without the need for a second surgical operation. In general, using manual DO methods in mandibular reconstruction is associated with the following complications: hypertrophic scarring, infection, relapse, nerve injuries, tooth injuries, inappropriate DV, device failure, and fusion error [21]. The existing complications in intra- and extra-oral DO devices show the need for the development of novel distractors using alternative techniques to reduce the negative effects and limitations of the existing DO methods.

The purpose of this study was to design and develop a two-axis automatic continuous distractor, with BB and TB end effectors, to be used in MRAs. In the proposed method, there are two linear mechanisms that can generate independent, continuous DFs. The generated DFs can be applied to the moving BS in the desired DVs. Both TB and BB distraction mechanisms can be connected to the proposed system to facilitate the application of the device in different reconstruction conditions. The positioning of the TB/BB end effectors is controlled by a high-precision control system. By using this technique, linear and curve-linear DVs can be customized and used in different reconstruction conditions. The control system of the device can precisely control the positioning of the moving BS in two DVs by executing two controlled DFs. By using the continuous DO method, a higher DR can be applied during the treatment, shortening the treatment time. Moreover, using the continuous DO technique improves the quality of the regenerated bone tissue. The proposed continuous DO device represents a novel approach to mandibular reconstruction that can meet the limitations of intra- and extra-oral DO devices for performing a successful DO with minimal side effects.

## 2. Materials and Methods

### 2.1. Design and Principles

The proposed two-axis automatic continuous distractor can be used in different MRAs for lengthening the mandible in different situations. The design of the system consists of different units that work together to generate two controlled DFs to be transmitted to the BB/TB end effectors, so that the DFs can be applied to the moving BS of the mandible. During the DP, there are different distraction factors that can significantly influence the bone regeneration/healing mechanisms, including DR, distraction rhythm, DF, and DV [5,15]. Therefore, a continuous DO device should be capable of executing controlled and smooth DF while moving the BS at the desired distraction rhythm/rate.

The proposed system consists of different units, including a power management system, rechargeable battery system, control system, mechatronic system, force transition system, and mechanical BB/TB end effectors. In the control system, a microcontroller is implemented to control the performance of the system while performing the DP. The power supply and power management units are used to supply the system with a regulated 5 VDC, and to recharge the batteries of the system. In the designed mechanism, a mechatronic system is used for executing separate controlled linear motions while generating two independent continuous pushing forces (i.e., DFs). In the mechatronic system, two stepper motors are used to execute two controlled angular motions with the desired parameters. The microcontroller can control the performance of the stepper motors using a precise open-loop control system. The control signals are transmitted to the motor drivers to drive the stepper motors. Each stepper motor is connected to a linear mechanism that can execute a controlled linear movement with high positioning accuracy. The generated force is transmitted to the BB or TB mechanical end effectors, which are installed on the distraction zone, using the transition mechanism. The designed system can generate two continuous pushing forces (i.e., DFs) with the desired parameters. The DFs and other process parameters can be set using a removable Human–Machine Interface (HMI), which is connected to the control system.

**Figure 5:**
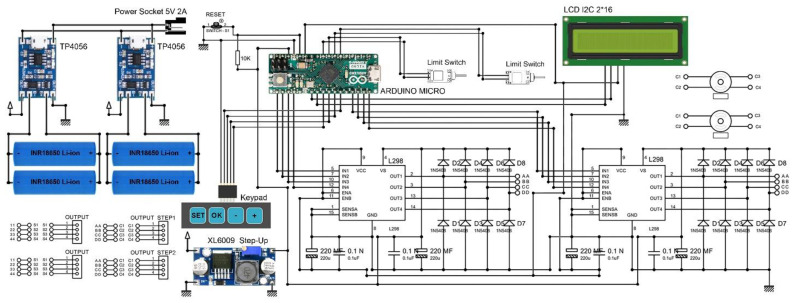
The detailed design of the control system.

Figure 5 presents the detailed design of the control system implemented within the proposed DO device. In the design of the control system, an Arduino Micro development board, based on the ATmega32U4 microcontroller, is used. This development board has a 16 MHz crystal oscillator and 20 digital input/output pins. The Arduino Micro has the required capabilities and working factors to be used for controlling the designed system. A 4-key keypad and a 2*16 Liquid Crystal Display (LCD) are used to set the process parameters, and to show/modify the working factors during the DP. The HMI unit is removable, and can be connected to the device before and/or during the DP. The DR and DL can be set/modified using the HMI unit. Two TP4056 battery chargers are connected to the power socket for recharging the lithium-ion batteries. The power management unit can recharge the batteries and supply different units, including the microcontroller, motor drivers, and display. By connecting the 8 digital pins of the microcontroller, the control signals for driving the stepper motors and the linear mechanisms are transmitted to two L298N dual full-bridge motor drivers. Two 28BYJ-48-12V (CenryKay, China) hybrid stepper motors are used in the designed system. The motor drivers can drive the stepper motors separately. An XL6009 DC–DC switch-mode boost step-up module is used to supply the L298 motor drivers with 12 VDC for driving the stepper motors.

A linear control method—Multi-Axis Automatic Controller (MAAC) [49,50]—is implemented within the control unit of the device. The MAAC is a precise linear control technique that can drive the stepper motors and execute linear motions with controlled parameters and high positioning accuracy. By using the MAAC control method, the linear mechanism can generate a smooth and continuous pushing force while executing various positioning rates and rhythms for performing the DP with the required distraction factors. The MAAC can drive the stepper motors in micro-stepping drive mode (1/32), with high accuracy and resolution. The micro-stepping drive mode can secure a soft and smooth positioning of the linear mechanism, enabling generation of a soft and smooth pushing force that is transmitted through the carriage of the linear mechanism. There are two limit switches implemented within the control system to define the start point (zero position) of the linear movement.

By using the HMI unit, two DLs can be set to define the desired positions of the end effectors in their linear vector. The DL defines the total travel of the end effector. The end effectors can move the BS in two predetermined linear DVs with independent DRs, which are usually equal. It is necessary to set the DR for each end effector using the HMI unit. The DVs can be customized using specific mechanical structures that can be connected to the end effectors. Subsequently, the control unit calculates the rhythm of the distraction steps based on the set DL and DR. In addition, the process/distraction time can be calculated in the control system and displayed on the HMI unit.

**Figure 6:**
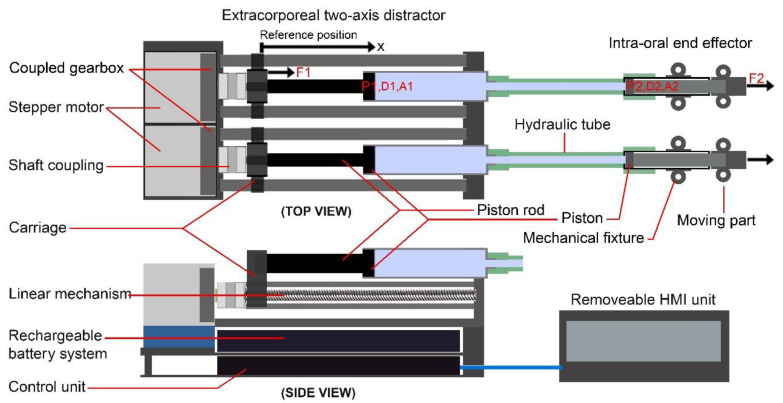
The schematic design of the mechanism for generating and transmitting two independent continuous forces with the end-effector-controlled positioning.

Figure 6 illustrates the working principles and the schematic design of the two-axis automatic continuous distractor. As illustrated in this figure, the stepper motor shaft is connected to the leadscrew of the linear mechanism using a solid shaft coupling. Therefore, when the control system drives the stepper motor, the rotational motion of the shaft is translated to linear movement of the carriage. Each carriage is fixed to the piston rod of a miniature hydraulic cylinder. This mechanism can apply the generated pushing force to the piston in a linear vector and transfer the generated force to the end effector using a flexible high-pressure hydraulic tube.

The 28BYJ-48 stepper motor has a step angle of 5.625 degrees. The stepper motor shaft is connected to a miniature gearbox with a gear–box ratio of 1/64. In each linear mechanism, a leadscrew of 3 mm diameter (M3), right-hand internal- and external-screw thread, with 0.5 mm pitch, and a length of 90 mm, is used. The stepper motor’s shaft is connected to the leadscrew by a 9 mm solid shaft coupling for translating the rotational movement to linear motion. Theoretically, by driving the stepper motor in micro-stepping drive mode (1/32), the shaft position changes by 0.0028°/step, and the carriage of the linear mechanism is moved 4 nm. A full rotation of the shaft moves the carriage of the linear mechanism 0.5 mm. By considering the set DR and DL as well as the positioning accuracy of the linear mechanism (4 nm/step), the control system can calculate and apply the desired distraction rhythm and distraction time for performing the DO process with the desired working factors.

The carriage of the linear mechanism is connected to the piston rod of a miniature hydraulic cylinder. The piston rod has a diameter of 5 mm and a length of 60 mm; the piston (P1) has a diameter of 8 mm and a length of 10 mm, and the cylinder body has a diameter of 12 mm with a length of 82 mm. When the linear movement is executed, the carriage pushes the piston rod, and the generated pushing force is applied to the piston of the hydraulic cylinder while pushing the hydraulic liquid inside the cylinder. The applied force is transferred to the other side of the transition system, which is the end effector. The end effector consists of a mechanical structure (intra-oral fixture) and a miniature hydraulic cylinder. The intra-oral miniature cylinder has a diameter of 8 mm and a length of 45 mm; the piston (P2) has a diameter of 6 mm and a length of 5 mm, and the piston rod has a diameter of 5 mm and a length of 30 mm. The piston rod is fixed to a mechanical fixture to fix the end effector to the BS. The transmitted pushing force pushes the piston of the intra-oral cylinder, and the piston rod is moved in a linear vector. By using this mechanism, the generated continuous force can be transmitted and applied to the moving BS at the desired DV. The transferred force can be calculated using the Pascal principle (Equation (1)):

(1) $$F_{1}\times \frac{D_{1}}{D_{2}}=F_{1}\times \frac{A_{2}}{A_{1}}=F_{2}$$

where *F*_1_ is the applied force to piston *P1*, *D*_1_ is the displacement of the carriage/piston *P1*, and *A*_1_ is the area of piston *P1*, while *F*_2_ is the applied force to piston *P2*, *D*_2_ is the displacement of the end effector/piston *P2*, and *A*_2_ is the area of piston *P2*. The control system can drive each linear mechanism with independent working parameters. Therefore, two controlled DFs can be applied to the moving BS.

By using Equation (1), the positioning accuracy of the end effector, as well as the amount of the transmitted pushing force, can be calculated. Piston *P1* has a diameter of 8 mm, while piston *P2* has a diameter of 6 mm. This means that when a linear movement is executed and the carriage of the linear mechanism and piston *P1* are moved 4 nm, piston *P2* and the end effector are moved 7 nm. Furthermore, when the pushing force *F*_1_ is applied to piston *P1*, it is transferred to the intra-oral cylinder and pushes piston *P2* with *F*_2_, which is connected to the end effector. For example, if a pushing force of 10 N is applied to piston *P1*, the transmitted force to piston *P2* equals 5.6 N.

### 2.2. Intra-Oral BB and TB End Effectors

**Figure 7:**
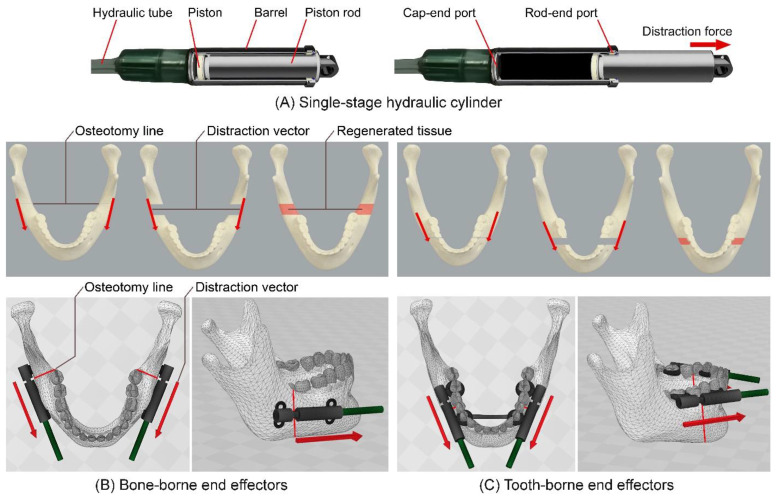
Intra-oral single-stage cylinder, and BB and TB end effectors for different MRAs: (**A**) intra-oral hydraulic cylinder; (**B**) BB end effectors; (**C**) TB end effectors.

It is important to adjust the intra-oral part of the distractor with the specific conditions of each patient and with the required treatment. Suitable intra-oral BB/TB end effectors should be adopted and used, as there are different treatment conditions in MRAs. As illustrated in Figure 7, two DVs are required in mandibular reconstruction for lengthening the mandible bone. In the manual reconstruction methods, the manual intra-/extra-oral distractors are used, two DVs are defined, and two manual forces are applied to the BS to generate the DF in the desired direction. In the proposed automatic system and method, two continuous DFs, with controlled DR, are applied to the BS in two predetermined linear vectors (DVs). The DVs can be modified in different treatment modalities. By setting the suitable DL and DR, the BS can be moved towards the desired position while the bone tissue is regenerating in the generated gap.

Two different types of end effectors (BB and TB) can be connected to the system. The design and structure of the end effectors depends on the patient and the reconstruction modality. Mechanical fixtures with different shapes, customized size, and predetermined DVs can be used according to the requirements of the specific reconstruction application. Two typical designs of the BB and TB end effectors are illustrated in Figure 7. As illustrated in this figure, in both BB and TB end effectors, a miniature single-stage hydraulic cylinder is used to produce linear actuation utilizing hydraulic pressure. The pressure of a hydraulic fluid pushes the piston in the desired direction. The generated and transmitted pushing force moves the piston rods, which are connected to the moving parts of the end effectors and push the BS in two distinct predetermined DVs. It can be seen that in the BB end effector is installed on the mandible, and the generated DFs are directly applied to the BS. In the TB end effector, the mechanism is dental-anchored, and the generated DFs are indirectly applied to the BS.

### 2.3. Mathematical Modeling

**Figure 8:**
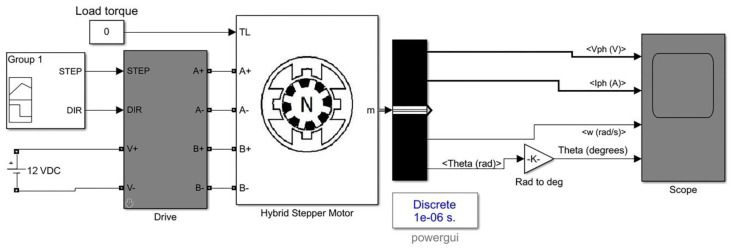
The mathematical model of the designed control system.

In the designed system, a precise linear control method (i.e., MAAC) is implemented within the control unit. The control unit can drive the hybrid stepper motors in micro-stepping drive mode. The designed control system and the hybrid stepper motors were modeled using MATLAB/Simulink software (MathWorks Inc., Natick, MA, USA). For evaluating the performance of the control system in controlling and driving the motor, the mathematical model of the stepper motor was designed. The designed model is illustrated in Figure 8, and consists of electrical and mechanical subsystems. The modeled control system was used to drive the hybrid stepper motor in micro-stepping drive mode with a controlled shaft positioning and speed. For the modeling of the stepper motor, differential equations of the stepper motor phases were used [51,52], as given below:

(2) $$L_{a}\frac{d_{i_{a}}\left(t\right)}{dt}=u_{a}\left(t\right)-e_{a}\left(t\right)-R_{a}i_{a}\left(t\right)$$

(3) $$e_{a}\left(t\right)=k_{m}\cdot \omega_{m}\cdot sin(Ƥ\theta_{m})$$

(4) $$L_{b}\frac{d_{i_{b}}\left(t\right)}{dt}=u_{b}\left(t\right)-e_{b}\left(t\right)-R_{b}i_{b}\left(t\right)$$

(5) $$e_{b}\left(t\right)=k_{m}\cdot \omega_{m}\cdot cos(Ƥ\theta_{m})$$

where *a* and *b* are the stepper motor phases, *R* is the phase resistance, *L* is the phase inductance, *u* is the terminal voltage, *e* is the back EMF, *θ_m_* is the angular position of the rotor, *ω_m_* is the angular speed of the rotor, *k* is the motor constant, and *Ƥ* is the number of stepper motor pole pairs.

After running the simulation, the scope of the model illustrates the simulated waveforms, including the phase voltage and phase current of the stepper motor phases, along with the rotor position and speed.

### 2.4. Experimental Study

**Figure 9:**
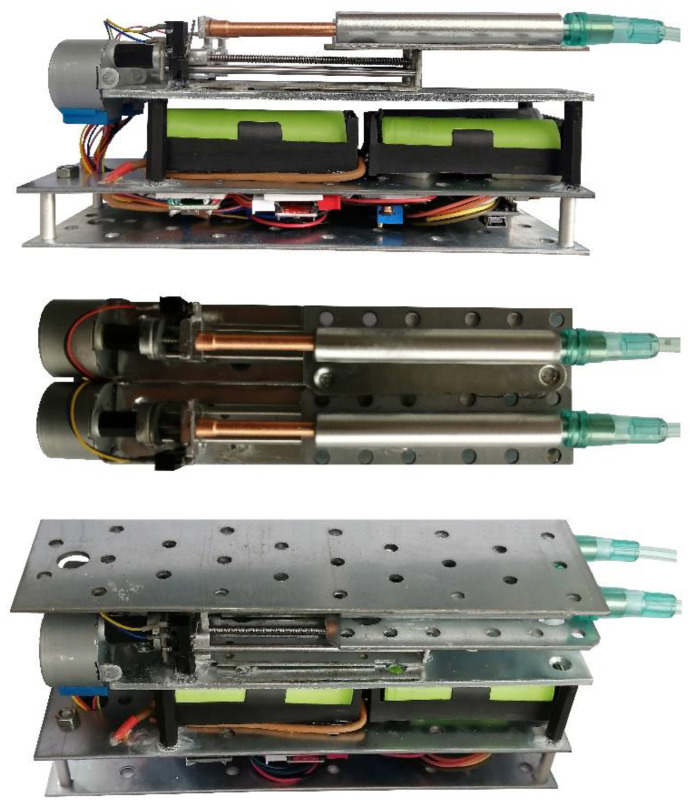
The first prototype of the two-axis automatic continuous distractor.

After the design of the proposed system, the device was prototyped. Figure 9 presents the first prototype of the two-axis automatic continuous distractor. The generated pushing force can be transmitted to the intra-oral end effectors using the hydraulic transition system with flexible tubes. The transmitted pushing forces are applied to the end effectors so that the BS can be moved in two predetermined linear vectors. After the design and development of the proposed system, experimental tests were performed to evaluate the performance of the control system and the functionality of the device in generating, transmitting, and applying the generated DFs for moving the BS in the desired DVs. In the first phase of the experimental study, the positioning of the linear mechanism was evaluated. In the second phase of the experiment, the generated and transmitted DF was measured. In the third phase of the experimental verification, the rechargeable battery system and the capacity of the batteries were examined.

## 3. Results

After the system design, modeling and experimental tests were performed to evaluate the performance of the control system in controlling the DP with desired working factors, and to justify the functionality of the device in generating two continuous DFs and performing continuous DO for reconstruction of mandibular deficiencies. In the following subsections, the results of simulation, experimental study, and battery system evaluation are presented and discussed.

### 3.1. Simulation Results

**Figure 10:**
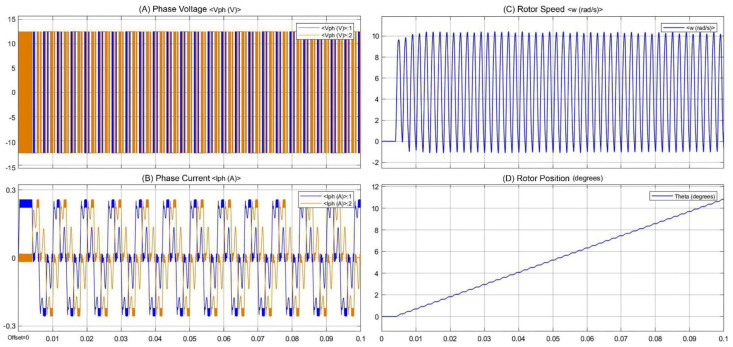
Simulation results of the modeled control system and stepper motor: (**A**) phase voltage; (**B**) phase current; (**C**) rotor speed; (**D**) rotor position.

After the modeling of the control system using MATLAB/Simulink software, the simulation was run for 0.1 s. The generated waveforms are presented in Figure 10. The simulation results include the phase voltage and phase current of the stepper motor’s phases, the rotor position, and the rotor speed. In the generated waveforms, *V*_1_ is the phase voltage of phase *1* and *V*_2_ is the voltage of phase *2* of the stepper motor. *I*_1_ is the phase current in phase *1* and *I*_2_ is the phase current of phase *2* of the stepper motor. It can be seen from the generated waveforms that the control system can drive the stepper motor with high positioning accuracy. A soft and continuous motion of the rotor can be seen, while the rotor speed and angular position are well controlled. Moreover, the generated voltage and current waveforms are similar cosine and sine waves, and are 90° displaced. The generated waveforms are in accordance with the theoretical equations of the stepper motor. It can be deduced from the obtained results that the designed control system can drive the stepper motor with high positioning accuracy, while the linear mechanism translates the rotation to linear motion with controlled working parameters. The simulation results justify the functionality of the designed control system in controlling the stepper motors with the required working factors.

### 3.2. Measurement of the Generated Force and Linear Positioning of the Mechanism

**Figure 11:**
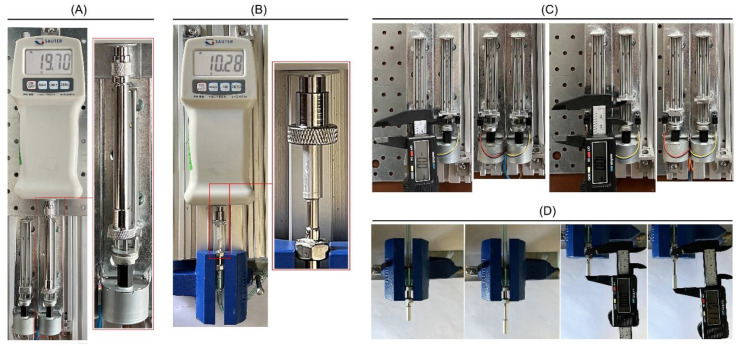
The experimental tests: (**A**) measuring the generated pushing force via linear mechanism; (**B**) measuring the transmitted force (i.e., DF); (**C**) linear positioning of the carriage of the linear mechanism; (**D**) linear positioning of the end effector.

The developed control system can generate two linear movements with controlled linear positioning and sufficient pushing force to move the end effectors of the system with high precision and sufficient DFs for moving the BS. The experimental evaluation had four phases. The experimental setup and the conditions of the tests are presented in Figure 11. In the first phase of the experiment, one of the linear actuators was used, and the device was set to perform a linear motion with a carriage movement rate of 3 mm/day, while the stepper motor was driven in micro-stepping drive mode, and the carriage of the linear mechanism was pushing the mechanical part of the force gauge, as illustrated in the Figure 11A. For measuring the generated pushing force, a SATUR FK50 digital force gauge with a resolution of 0.02 N was used. The linear mechanism can generate a pushing force of 19.70 N for pushing the piston rod of the hydraulic cylinder.

In the second phase of the experimental test, the transmitted force (i.e., DF) to the end effector was evaluated, as illustrated in Figure 11B. In this phase, one of the intra-oral end effectors was used. The device was set to perform different standard DPs with a DR of 3 mm/day and DL of 15 mm, while the stepper motor was driven in micro-stepping drive mode. Theoretically, according to Equation (1), when a pushing force of 19.70 N is generated, 11.08 N should be transmitted to the end effector. The result of this force measurement test shows that a DF of 10.28 N was delivered to the end effector.

In the third and fourth phases of the experimental study, the positioning accuracy of the linear mechanism and the end effector were evaluated, as illustrated in Figure 11C,D, respectively. The carriage of the linear mechanism was set at the start position. Subsequently, the system was run while performing DPs using different working factors (Table 1). After the completion of each test, the total travel of the carriage was measured, showing the positioning accuracy of the linear mechanism. The travel length of the carriage was measured with a digital caliper, with a resolution and accuracy of 0.01 mm. Moreover, one of the end effectors of the device was fixed while the device was performing DPs with different working conditions. The total travel of the end effector (i.e., DL) was measured after the completion of each DP. According to the results of the experimental study, as presented in Table 1, the developed two-axis distractor can control the linear position of the carriage of the linear mechanism with a high positioning accuracy (<100 µm), a low positioning error in total travel (<100 µm), and a mean carriage positioning error rate of 0.2% of the desired travel length. Furthermore, the transmitted force can push and move the moving part of the intra-oral end effector with a high positioning accuracy (<1 mm), a low positioning error in the executed DL (<1 mm), and a mean end effector positioning error rate of 2.7% of the desired DL.

### 3.3. Evaluation of the Battery System

In the last phase of the experimental study, a discharge test was performed on the batteries to evaluate the capacity of the designed battery system. In addition, the functionality of the rechargeable battery system in providing the required energy for running the system was evaluated. In this experiment, four INR18650 3.7 V Li-ion cells, with a capacity of 2500 mAh and a cutoff voltage of 2.7 V, were used. The batteries underwent evaluation using a Bitrode MCV 16–10 low-current life-cycle cell tester. The battery cells were connected in two series sets; each set consisted of two parallel battery cells. In this experiment, the cutoff voltage was set to 5 VDC, while the discharge procedure was performed at 1 A. The discharging procedure continued until the cutoff voltage was reached. 

**Figure 12:**
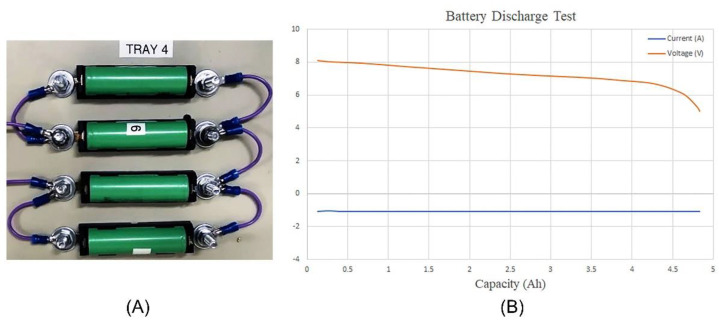
Battery discharge test: (**A**) the test procedure; (**B**) the obtained results.

Figure 12 presents the test condition and the results of the battery discharge test, including the combination of cells, along with the current-versus-capacity and voltage-versus-capacity graphs. The battery cells discharged for 4.5 h at a current of 1 A. In the discharge process, the voltage of the battery cells dropped from 8.2 VDC (fully charged) to a cutoff voltage of 5 VDC. It can be seen in the generated current-versus-capacity graph that there was no current spike during the discharge test, which means that the battery cells were in good health and combination, with no temperature increase or short-circuit. The results of the battery discharge test show that the designed battery system and the selected battery cells, with a total capacity of 4.85 Ah, are suitable to be used in the device for supplying the system and performing the DP.

By considering the power consumption of each component that is used in the system, the power consumption of the device can be calculated. The power consumption of the device is approximately 1000 mAh. Therefore, the battery system should be capable of supplying the device for 4.5 h before the need for a recharge. After the batteries were fully charged, the system was run while the capability of the battery system in supplying the required energy under different working conditions was monitored. The battery system could supply the system with required voltage and current for up to 4 h before reaching the cutoff voltage.

## 4. Discussion

DO is a promising surgical technique for the reconstruction of bone deficiencies in different body zones. The application of DO in the reconstruction of mandibular deformities has shown promising results compared to conventional methods for mandibular reconstruction. However, there are limitations associated with manual DO devices, including the size of the device, scar formation, manual operation, low accuracy/repeatability, unknown DF, and intermittent activation of the distractor [5]. The existing limitations in manual distractors limit their application in MRAs. DR, distraction rhythm, DV, DF, repeatability, resolution, and accuracy are the important parameters that can be improved to enable an ideal solution to be used in MRAs.

Recently, the application of continuous distractors in MRAs has shown better treatment conditions and outcomes compared to manual DO methods. The continuous distraction of the bone enables the application of higher DRs during the DP without negatively affecting the bone regeneration/healing mechanisms. Applying a soft and smooth DF can enhance the quality of the regenerated bone tissue. Thus, by using a continuous DO method, the treatment time can be significantly reduced. Using an automatic method can also reduce the risks and complications during and after the treatment [5,15,53]. Two types of system have been implemented within the design of automatic distractors for generating a continuous force: motor-driven systems, and hydraulic systems. In the motor-driven distractors, the continuous force is generated using a motor-based mechanism. In the hydraulic distractors, the continuous force is generated using a hydraulic system. The motor-driven systems have limitations in transmitting the generated DF, while hydraulic systems have limitations in generating a soft and continuous DF [5]. In addition to the limitations of these techniques in generating and transmitting a controlled, soft, and continuous DF, the existing systems have other limitations, including the size of the distractor and the DV. The existing continuous distractors have only one DV, as they can control only one axis, which limits their application in MRAs. There is still a gap between the existing technologies and an ideal device for performing continuous DO in MRAs.

**Figure 13:**
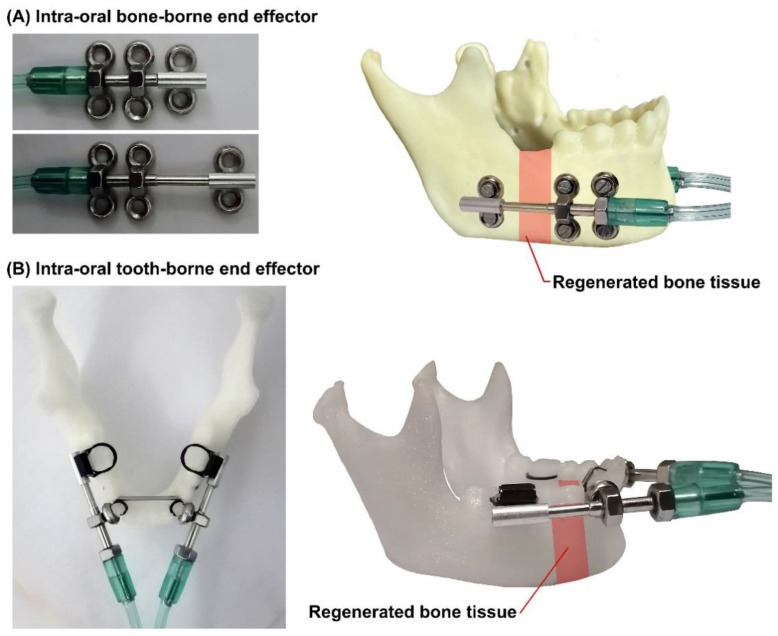
Illustration of the application of two-axis continuous distractor in MRAs using mandible models: (**A**) intra-oral BB end effectors; (**B**) intra-oral TB end effectors.

In the proposed device, a two-axis hybrid mechanism, combining motor-driven and hydraulic techniques, is used to execute two controlled, soft, and steady motions that can generate two independent, smooth, and continuous pushing forces. The generated forces are transmitted to the end effectors using a miniature transition system, including two flexible high-pressure hydraulic tubes. The end effectors apply two continuous DFs to the BS in two independent linear DVs. The device can deliver two independent DFs of 10.28 N to the BB/TB end effectors, while controlling the linear positioning of the end effector with a positioning error less than 1 mm. The DVs can be set and customized using the mechanical structure (design) of the BB/TB end effectors. The results of modeling and experimental study show that the developed system can perform the continuous distraction procedures with a controllable rate and rhythm, high positioning accuracy, and controlled DF. The battery system makes the device portable, while supplying the device for up to 4 h before the need for a recharge. The removable HMI unit makes the device user-friendly, and enables setting/modification of the working factors of the system—including the distraction parameters—before/during the DP. Figure 13 illustrates the application of the proposed two-axis continuous distractor in MRAs.

The device can perform standard DO protocols under different working conditions, with controllable DR and DL, while applying two continuous DFs for moving the BS in two DVs. By using two different types of intra-oral mechanical fixtures for installation of the end effectors—namely, BB and TB end effectors—the device can be used for treating different mandibular reconstruction conditions. By using the intra-oral BB end effectors, the DF can be directly applied to the BS. However, using intra-oral BB end effectors is associated with limitations such as scar formation, nerve injuries, patient discomfort, and other complications [1,27,48].

The application of TB end effectors is less complicated compared to intra-oral BB end effectors. Installation and removal of the intra-oral TB end effectors require no surgical operation; only one surgical operation is required for the mandibular osteotomy. However, the application of TB end effectors is limited, and only small DLs can be executed using the TB end effectors. Moreover, when a TB end effector is used, the DF is applied to the tooth, and the applied DFs cannot completely be transferred to the BS. This can cause the teeth to move, as the DF induces bone resorption on the pressure side and bone apposition on the tension side, while reducing the positioning accuracy of the BS during the reconstruction process. The application of intra-oral BB end effectors is more predictable compared to that of TB end effectors. In the proposed system, both end effectors can be connected to the system for performing a reconstruction process using the suitable type of intra-oral end effector. By using the suitable type and customized size of the end effectors, different mandibular deficiencies—including mandibular growth defects, mandibular retrognathia, hemifacial microsomia, and localized bone loss—can be reconstructed.

## 5. Conclusions

The proposed device is a novel two-axis automatic continuous distractor that can be used in the reconstruction of mandibular deficiencies. The device enables an automatic DP while generating two continuous forces for moving the BS in two independent DVs. The desired DVs can be set precisely, and the BS can be moved to the desired position in an automatic and fully controlled distraction procedure.

By using the proposed two-axis distractor, the positioning of the end effectors, as well as the applied forces to the BS, is fully controllable during the DP. By using BB and TB end effectors, using continuous DO methods in reconstruction applications with different conditions can be possible. Using a continuous distraction method can lead to higher DRs applied to the BS, while regenerating the bone tissue with higher quality in a shorter treatment time. This method meets the limitations of the existing intra- and extra-oral DO methods, while improving the treatment conditions. In future studies and possible clinical trials, the two-axis continuous distractor could be used as an ideal solution in MRAs in humans.

## 6. Patents

A patent was produced from the research work reported in this manuscript. The method of continuous force generation and the force transition mechanism are under the protection of Taiwan’s Intellectual Property Office, as an invention patent, with publication/patent number M604182, application number 109209126, entitled: “Hybrid traction device for oral and maxillofacial reconstruction”.

## Figures and Tables

**Table 1:** Measurement of the linear positioning and the generated force.

Phase 1: Extracorporeal Linear Mechanism					
Test	Repeat Cycle	Carriage Movement Rate (mm/day)	Desired Carriage Travel (mm)	Mean Measured Carriage Travel (mm)	Mean Carriage Positioning Error Rate (%)
1	5	3	5	5.01	0.20
2	5	3	10	10.04	0.40
3	5	5	10	10.03	0.30
4	5	3	15	15.03	0.2
5	5	5	15	15.02	0.13
**Phase 2: Intra-Oral End Effector**					
**Test**	**Repeat Cycle**	**DR (mm/day)**	**Desired DL (mm)**	**Mean Measured DL (mm)**	**Mean DL Error Rate (%)**
1	5	3	5	5.23	4.6
2	5	3	10	10.26	2.6
3	5	5	10	10.18	1.8
4	5	3	15	15.23	1.5
5	5	5	15	15.46	3

## Data Availability

The research data related to this work are included within the manuscript. For more information on the data, contact the corresponding authors.
